# Identical injuries in 2 sisters (victims of motor vehicle collision): Two cases report

**DOI:** 10.1016/j.ijscr.2019.11.046

**Published:** 2019-11-27

**Authors:** Muhammad Elsayed Mahmoud, Khaled Zamel Aldaraan, Mohamed Hany Hassab

**Affiliations:** a Pediatric Surgery Department, Prince Mohammed bin Abdulaziz Hospital (PMAH), Riyadh, Saudi Arabia; b Pediatric Surgery Department, Faculty of Medicine, Al-Azhar University, Cairo, Egypt

**Keywords:** bpm, beat per minute, CT, computerized tomography, DMSA, dimercaptosuccinic acid, EMS, Emergency medical service, ICU, Intensive care unit, IV, Intravenous, MOF, Multi-organ failure, MVC, Motor vehicle collision, PALS, Pediatric advanced life support, RBCs, Red blood cells, U/S, Ultrasonography, Motor vehicle collision, Pediatric polytrauma, Renal trauma, Conservative management

## Abstract

**Background:**

Children sometimes are victims of motor vehicle collision (MVC) along with their families. Motor vehicle collision can cause several patterns of blunt trauma resulting from the impact itself or penetrating trauma by broken glass or other sharp objects. Most cases can be managed conservatively as long as the hemodynamic parameters are stable & hollow viscera are intact. Usually, cases shared the same accident have different injury patterns. But herein, we present 2 sisters as victims of MVC with same lesions but at variable degrees.

**Case presentation:**

Two sisters were referred to our hospital as victims of MVC and presented by nearly identical injuries (left lung injury, left kidney injury, retroperitoneal fluid collection, & left humerus fracture). Both girls were managed non-operatively until improved and became ready for discharge from the hospital in stable good health status.

**Conclusion:**

Identical injuries in pediatric trauma victims do occur and should be considered and reported to raise the awareness about the importance of submission to vehicle safety protective rules especially for children.

## Highlights

• Identical injuries in trauma pediatric victims shared the same trauma are not commonly encountered.
• This pattern of identical injury does occur and should be considered and reported.
• The aim is to raise the awareness about the importance of submission to vehicle safety protective measures especially for children.
• None of the injuries of this study cases mandated immediate surgical intervention. Even major renal trauma in pediatrics can be managed non-operatively as long as the patient is hemodynamically stable and serial radiologic studies showed improvement and gradual resolution of lesions.

## 1 Introduction

Pediatric blunt trauma is a relatively common occurrence representing one of the causes of preventable trauma-related morbidity and mortality. Up to 8% of bluntly traumatized children have abdominal injuries, primarily involving the solid viscera [1].

During the past two decades, management of blunt injury to abdominal organs has changed from operative to selective non-operative approach. Nowadays with the advances of trauma protocols, pediatric intensive care, and diagnostic/interventional radiology, it is reported that non-operative management of blunt injuries can reach a success rate of <95%. About 98.5% of patients with blunt hepatic injury received this management mode and successfully recovered. A similar success rate is reported for pediatric blunt non-vascular renal injury. Blunt splenic injury managed non-operatively has a reported success rate higher than 95% in pediatric patients [2,3].

Improved understanding of the natural history of splenic, hepatic, and renal injury significantly modified the management of blunt abdominal trauma in children. Mandatory laparotomy for proven or suspected hemoperitoneum became obsolete procedure that has been replaced by intensive observation of most patients who are stable or can be stabilized in the Emergency Department. This has led to a significant decrease in morbidity due to anaesthetic complications, post-splenectomy sepsis, and post-operative adhesions. To benefit from all these advances, physicians/surgeons caring for traumatized children should be aware of the often subtle manifestations of abdominal trauma, the available diagnostic modalities, and the requirements for early volume resuscitation [4].

To our knowledge, no previous cases of identical injuries in pediatric trauma have been reported. This is due to blind, unplanned haphazard nature of trauma resulting usually in diverse models of trauma patterns of injured organs. Herein, we present our report on identical injuries in 2 trauma victim sisters who were non-restrained in the back seat. They sustained motor vehicle collision (MVC) with their parents when their car hit an advertising column on the road [the parents died immediately at the trauma scene before approach of emergency medical service (EMS)].

The work has been reported in line with the SCARE criteria [14].

## 2 Case presentation

Two sister girls presented to our hospital with identical injuries as a result of MVC. They were non-restrained in the back seat. Both cases have been resuscitated and stabilized as per pediatric advanced life support (PALS) protocol guidelines.

## 3 Case 1

**Fig. 1:**
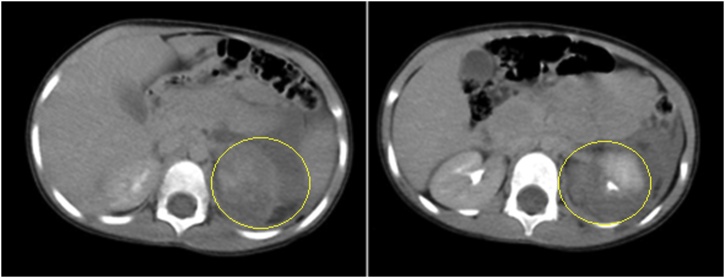
Case 1, CT trauma of the revealed shattered left kidney with huge perinephric and retroperitoneal hematoma (yellow circle).

The younger girl (3.7 years old) primary survey revealed that she was irritable, intact patent airway, equal bilateral air entry, tachycardic (144 beat per minute bpm), mildly hypotensive (110/40 mmHg, normal age-adjusted diastolic blood pressure is 46). Secondary survey showed only scalp laceration, with no bruises on the anterior or posterior abdominal wall. She had gross hematuria for which she had been catheterized. Hematuria gradually subsided until disappeared completely by 4th day of admission. Non-enhanced computerized tomography CT brain and maxillofacial scan revealed small hemorrhagic area at left frontal lobe with surrounding edema suggestive of hemorrhagic contusion & no midline shift. Also, posterior falcine and right tentorial hematoma & comminuted fracture of the right mandibular condyle are noted. CT trauma scan revealed multiple lung contusions at posterior aspect of left lower lobe, non-vascularized left kidney except small part at mid-zone and left perinephric and retroperitoneal hematoma (Fig. 1). At delayed phase, no excretion of contrast except from the vascularized part. These data suggest severe (grade IV) injury with shattered left kidney & devascularisation of most of its tissue. Small filling defect is noted at proximal part of left renal vein (denoting a small thrombus).

**Fig. 2:**
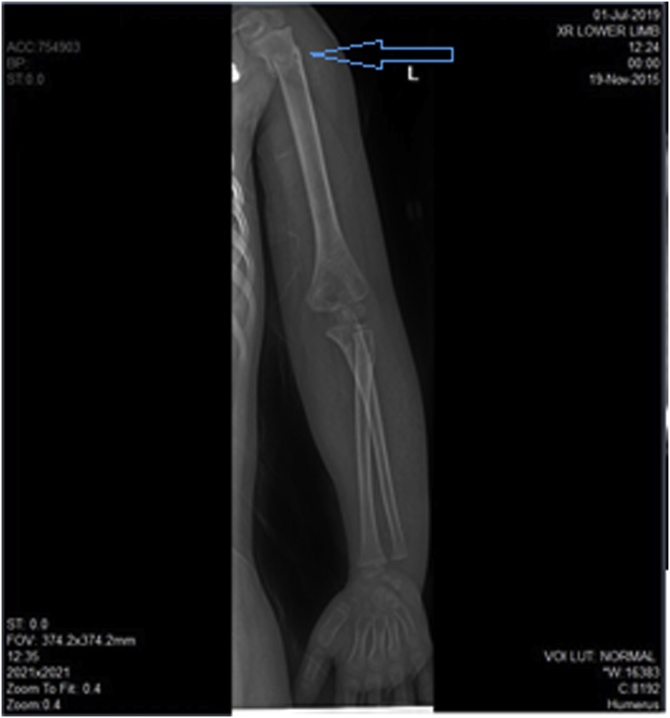
Case 1, plain x-ray left upper limb demonstrated fracture left humerus proximal shaft (blue arrow).

Multiple liver lacerations and moderate hemoperitoneum are noted. Radiographic studies revealed fracture left humerus proximal shaft (Fig. 2).

**Fig. 3:**
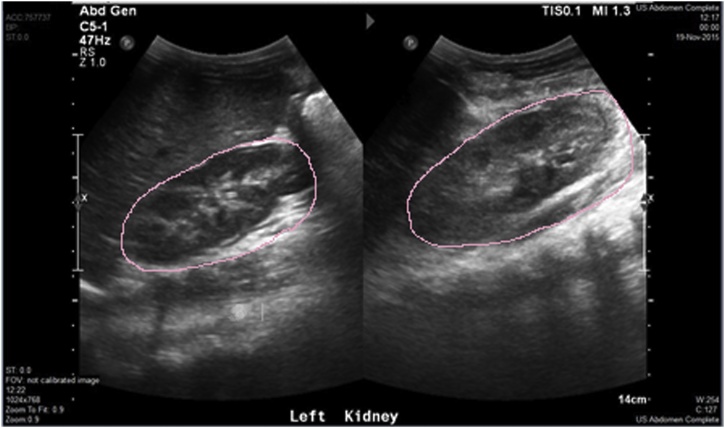
Case 1, repeated US of left kidney 2 months post-trauma revealed resolution the perinephric retroperitoneal hematoma & re-appearance of most of the calyceal system of the left kidney indicating significant improvement and healing (pink mark).

She was resuscitated by IV fluids and packed RBCs, admitted, and managed conservatively as regard the left renal and other visceral injuries. She had left arm below elbow cast for the fracture left humerus. In the setting of severely injured non-vascularized left kidney, many surgeons would rush to do nephrectomy, but we adopted the conservative management to give this kidney a chance to recover and heal. She improved dramatically in response to this mode of care [the renal parenchyma as well as the collecting system significantly healed as evidenced by repeat U/S 2 months later that revealed re-appearance of the calyces & the normal reniform shape denoting restoration of ∼60% of the renal architecture] (Fig. 3). Also 4 months later, renal dimercaptosuccinic acid (DMSA) scan was done and revealed differential right-to-left renal function of 62%-to-38% respectively. Interestingly, she did not develop hypertension, urinoma, renal vein thrombosis or renal artery aneurysm.

## 4 Case 2

**Fig. 4:**
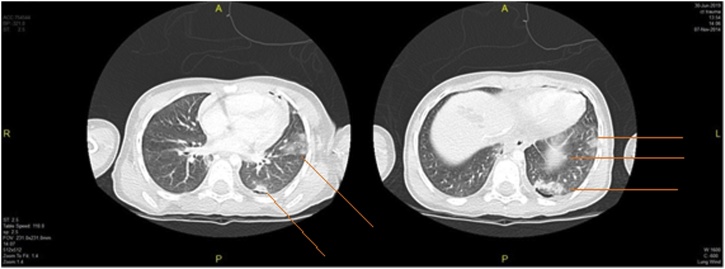
CT trauma revealed left lung contusions (orange lines).

**Fig. 5:**
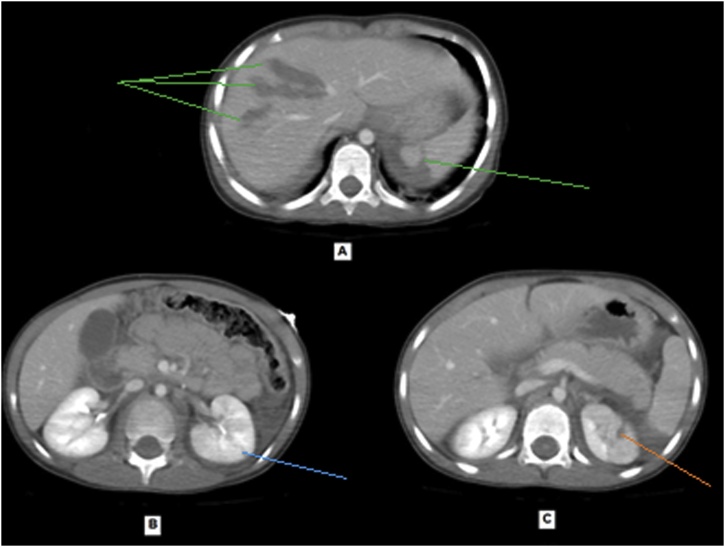
Case 2, CT trauma revealed A, multiple liver lacerations and splenic laceration (green lines). B, left renal laceration at upper zone (blue line) and C, left renal laceration at mid zone (orange line).

**Fig. 6:**
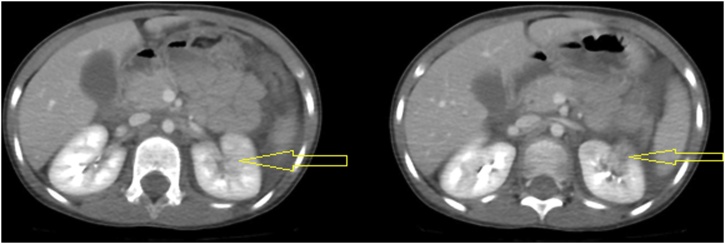
Case 2, CT trauma revealed left renal laceration and contusion (yellow arrows).

**Fig. 7:**
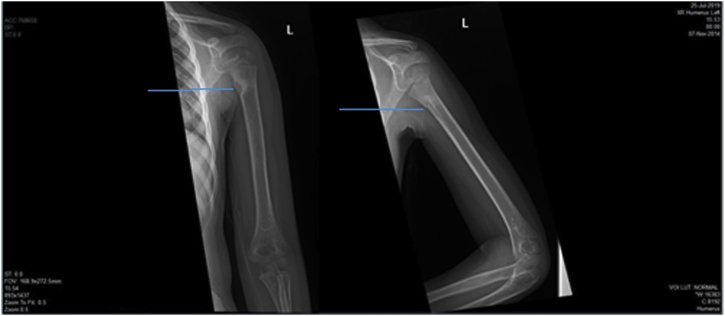
Case 2, plain x-ray left upper limb of the older girl revealed green-stick fracture of medial cortex left humerus surgical neck (blue lines).

The second girl (5 years old) primary survey revealed that she was calm, intact patent airway, equal bilateral air entry, tachycardiac (128 bpm), mildly hypotensive (112/42 mmHg, normal age-adjusted diastolic blood pressure is 46). Secondary survey was unremarkable. She had same injury to left lung, left kidney, and left humerus (but at milder degree) in addition to liver, & spleen lacerations. She had no hematuria. CT brain was normal. CT trauma pan scan revealed multiple left lung contusions & laceration at left lower lobe (Fig. 4) with mild left pneumothorax, mild pericardial effusion, multiple liver lacerations, splenic laceration associated with devascularisation of its posteromedial part (Fig. 5), marked hemoperitoneum, multiple small left renal lacerations are noted at upper, mid, and lower zones some reaching till the sinus suggesting grade II left renal injury (Fig. 6). Also, multiple wedge-shaped areas of hypo-attenuation suggestive of left renal contusions are noted. Radiographic studies revealed greenstick fracture of only the medial cortex of left humerus surgical neck (Fig. 7).

## 5 Discussion

Blunt trauma constitutes about 90% of pediatric trauma cases with the abdomen affected in approximately 8% of them. The kidney is injured in about 10% of victims. The primary goals of renal injury management are to preserve functional renal parenchyma and to minimize patient morbidity [5,6].

Expectant management of grade IV traumatic renal injury in children is safe and effective and offers the patients an excellent chance to achieve the primary goals of renal injury management [6].

Stable pediatric trauma patients spend less time in the hospital, return to school upon discharge, and are allowed lower hemoglobin levels prior to transfusion. Intensive care units (ICUs) are reserved for children with recent or ongoing bleeding, previously unstable, or with concomitant injuries necessitating ICU admission. On the contrary, operative management of bowel & pancreatic injuries gives more favourable outcomes [7].

Pediatric polytrauma is relatively common and requires individualized decision-making with the use of expertise from many specialities. There are many anatomical and physiological differences that in the context of polytrauma confirm the old axiom: ‘children are not small adults!’ [8].

Thanks to their malleable growing dynamic tissues, children have remarkable resilience and can make dramatic recoveries from seemingly irrecoverable situations. Injury patterns may also be different from the same mechanism due to relative size differences [8].

More than 90% of solid organ injuries in children can be treated conservatively and interpretation of images by experienced clinicians is vital to make proper decisions in correlation with the clinical data [8].

The concept for application of damage control strategy and early appropriate resuscitation in adults is to avoid the inflammatory cascade that occurs after trauma, leading to a robust systemic inflammatory response and the initiation of a sequential multi-organ failure (MOF) at around 48 hours post-injury. In children the response is different, often with a much smaller systemic inflammatory response and a more local inflammatory response. In children, MOF occurs early and simultaneously and often improves after prompt appropriate resuscitation [9,10].

Proponents of adoption of the well-established conservative treatment of blunt abdominal trauma include hemodynamic parameters, clinical status, and CT findings rather than the severity of injury. This is definitely successful even for pediatric patients with hemoperitoneum, altered sensorium, or higher grades of injury. Close surveillance in pediatric ICU is always desirable at least for the first days of admission to facilitate early detection of any deterioration [11,12].

Non-operative management of mild-to-moderate renal trauma (grades I to III) has become the standard of care provided that the injury is properly assessed & graded. This mode of management is successful for contusions, contained lacerations, and even lesions with moderate amount of hemat/urinoma in the vitally stable patient [12].

As regard radiologic follow up of pediatric cases with renal injury, it depends on ultrasonography not CT as recent studies of low-dose radiation CT in pediatric patients suggest that ionizing radiation life time cancer risk increases proportionately with decreasing age and increasing dose of radiation [13].

Lastly, the girls need a great psychosocial support and this exactly what their relatives understand and try to cope with in order to compensate the space resulting from the girls' parents loss.

## 6 Conclusion

Identical injuries in trauma pediatric victims shared the same trauma are not commonly encountered. None of the injuries of the fore-mentioned cases mandated immediate surgical intervention. Even major renal trauma in pediatrics can be managed non-operatively as long as the patient is hemodynamically stable and serial radiologic studies showed improvement and gradual resolution of lesions. Also, vehicle safety protective measures may need to be revised as regard design, stability, seat size in relation to child's body size, safety belt direction or orientation.

## Funding

There are no sources of funding to acknowledge.

## Ethical approval

Our study exempted from ethical approval.

## Consent

Written informed consent was obtained from the patient's parents for publication of this case report and accompanying images. A copy of the written consent is available for review by Editor-in-Chief of this journal on request.

## Author contribution

Khaled Z. Aldaraan – Conception of the work, Design of the work, Drafting the work, Revising the work critically for important intellectual content, Final approval of the version to be published, Agree to be accountable for all aspects of the work in ensuring that questions related to the accuracy or integrity of any part of the work are appropriately investigated and resolved.

Muhammad E. Mahmoud – Conception of the work, Design of the work, Drafting the work, Revising the work critically for important intellectual content, Final approval of the version to be published, Agree to be accountable for all aspects of the work in ensuring that questions related to the accuracy or integrity of any part of the work are appropriately investigated and resolved.

Mohamed H. Hassab – Conception of the work, Design of the work, Drafting the work, Revising the work critically for important intellectual content, Final approval of the version to be published, Agree to be accountable for all aspects of the work in ensuring that questions related to the accuracy or integrity of any part of the work are appropriately investigated and resolved.

## Registration of research studies

None.

## Guarantor

The corresponding author is the guarantor of submission.

## Provenance and peer review

Not commissioned, externally peer-reviewed.

## Declaration of Competing Interest

There is no conflict of interest to disclose.

## References

[1] Keller M.S. (2004). Blunt injury to solid abdominal organs. Semin. Pediatr. Surg..

[2] Thompson S.R., Holland A.J. (2006). Current management of blunt splenic trauma in children. ANZ J. Surg..

[3] Yanar H., Ertekin C., Taviloglu K., Kabay B., Bakkaloglu H., Guloglu R. (2008). Nonoperative treatment of multiple intra-abdominal solid organ injury after blunt abdominal trauma. J. Trauma.

[4] Schiffman M.A. (1989). Non-operative management of blunt abdominal trauma in pediatrics. Emerg. Med. Clin. North Am..

[5] Peclet M.H., Newman K.D., Eichelberger M.R., Gotschall C.S., Guzzetta P.C., Anderson K.D., Garcia V.F., Randolph J.G., Bowman L.M. (1990). Patterns of injury in children. J. Pediatr. Surg..

[6] Umbreit Eric C., Routh Jonathan C., Husmann Douglas A. (2009). Non-operative management of non-vascular grade IV blunt renal trauma in children: meta-analysis and systematic review. Pediatr. Urol..

[7] Notrica D.M. (2015). Pediatric blunt abdominal trauma, current management. Curr. Opin. Crit. Care.

[8] Peterson N., James J. (2018). Polytrauma in children. Orthoped. Trauma.

[9] Rankin J.A. (2004). Biological mediators of acute inflammation. AACN Clin. Issue.

[10] Pandya N.K., Upasani V.V., Kulkarni V.A. (2013). The pediatric polytrauma patient: current concepts. J. Am. Acad. Orthop. Surg..

[11] McConnell D.B., Trunkey D.D. (1990). Non-operative management of abdominal trauma. Surg. Clin. North Am..

[12] Ghimire P., Ghimire P., Yogi N. (2013). Non-operative management of blunt abdominal trauma in a tertiary care hospital of Nepal. Nepal J. Med. Sci..

[13] Brody A.S., Frush D.P., Huda W., Brent R.L. (2007). American Academy of Pediatrics Section on Radiology: radiation risk to children from computed tomography. Pediatrics.

[14] Agha R.A., Borrelli M.R., Farwana R., Koshy K., Fowler A., Orgill D.P., For the SCARE Group (2018). The SCARE 2018 statement: updating consensus surgical CAse REport (SCARE) guidelines. Int. J. Surg..

